# Assessment of central corneal thickness and corneal endothelial morphology using ultrasound pachymetry, non-contact specular microscopy, and Confoscan 4 confocal microscopy

**DOI:** 10.1186/1471-2415-13-73

**Published:** 2013-11-25

**Authors:** Haya Matuoq Al Farhan, Wafa’a Majed Al Otaibi, Hanouf Mohammed Al Razqan, Alanoud Abdullah Al Harqan

**Affiliations:** 1 Department of Optometry and Vision Sciences, College of Applied Medicine Sciences, King Saud University, P.O. Box 10219, Riyadh 11433, Kingdom of Saudi Arabia

**Keywords:** Corneal endothelial morphology, Central corneal thickness, Ultrasound pachymetry, Non-contact specular microscopy, Confocal microscopy, Reproducibility, Repeatability, Inherent precision

## Abstract

**Background:**

The aim was to compare the repeatability, reproducibility and inherent precision of ultrasound pachymetry (USP), noncontact specular microscopy (SP-2000P) and the Confoscan 4 confocal microscope (z-ring CS4) in measuring endothelial cell density (ECD), coefficient of variation of cell size (CV), and central corneal thickness (CCT) in normal eyes.

**Methods:**

In this prospective study, one eye was selected from each of 30 subjects for the measurements of ECD, CV and CCT, which were taken by two observers. Results were analyzed statistically by repeated-measures analysis of variance (ANOVA) for intra-observer repeatability, inter-observer reproducibility, unpaired *t*-test, paired *t*-test, and Bland–Altman analyses to determine limits of agreement (LOA) between the three instruments.

**Results:**

Mean ECD, measured by SP-2000P and z-ring CS4, were 3115.50 ± 279.70 cells/mm^2^ and 3167.50 ± 264.75 cells/mm^2^, respectively (observer 1), and 3192.63 ± 249.42 cells/mm^2^ (z-ring, observer 2). Mean CV measurements were 27.12 ± 2.51 and 27.10 ± 2.41 (SP-2000P and z-ring CS4, respectively; observer 1), and 27.17 ± 2.25 (z-ring, observer 2). Mean CCT values were 555.11 ± 35.83 μm (USP), 535.82 ± 41.10 μm (SP-2000P) and 552.57 ± 36.83 μm (z-ring CS4), and 554.97 ± 36.34 μm (z-ring CS4, observer 2). However, pairwise tests in all cases there was good repeatability and reproducibility as shown by inter-observer and intra-observer analysis of variance for each of the instruments.

**Conclusions:**

The SP-2000P and the z-ring CS4 can be used interchangeably to measure ECD and CV. For CCT, the sample size was too small to test for differences of the CCT measurements between the three instruments.

## Background

Corneal endothelial morphology and central corneal thickness (CCT) are important parameters for evaluating the cornea; particularly in the case of post-refractive surgery assessment [1-3]. Key corneal endothelial morphology parameters include the endothelial cell density (ECD), and the coefficient of variation of cell area (CV/polymegethism). Both of these measures can be affected by a broad range of disorders, such as contact lens complications [4,5], glaucoma [6,7], dry eye [8], and diabetes mellitus [9,10]. Furthermore, it is predictable that a normal healthy endothelium will have a low CV value [11].

The conventional method to estimate ECD is by using slit-lamp biomicroscopy [12,13]; however, a disadvantage of this technique is that it is a manual assessment that requires subjective interpretation by the observer [14]. Several automated instruments have been introduced to objectively evaluate corneal endothelial morphology and CCT. These include the Topcon Optical SP-2000P non-contact specular microscope (SP-2000P) [15,16], the Confoscan 4 confocal microscope (CS4) [17,18], and a non-contact specular microscopy system, Topcon SP-3000 [18,19].

The handheld ultrasound pachymeter (USP) is the most commonly used instrument for measuring CCT. However, studies report that the CCT measured with the USP can significantly vary between examiners [20,21]. Problems with reproducibility can be due to inaccurate alignment of the probe and when the probe is laid obliquely on the cornea—errors that can lead to thicker measurements of CCT [22,23]. Recently, several newer instruments have become available to measure the CCT such as the Visante™ anterior segment optical coherence tomography (Visante OCT) [24], CS4 [14], ultrasound biomicroscopy (UBM) [25], and SP-2000P [15].

A number of studies have made comparisons of ECD, CV, and CCT measurements of the SP-2000P [26,27], CS3 [28], and z-ring CS4 [29]. To the best of our knowledge, this is the first study to compare the ECD, CV, and CCT measurements obtained with USP, SP-2000P and z-ring CS4 on normal eyes.

The purpose of this study was to compare the inherent precision of CCT measurements using USP, SP-2000P, and z-ring CS4, based on intra-observer repeatability, and to assess the inter-observer reproducibility using the z-ring CS4. We also aimed to assess the intra-observer repeatability of ECD and CV measured using SP-2000P and z-ring CS4, and the inter-observer reproducibility using the z-ring CS4.

## Methods

This prospective study assessed 30 eyes of 30 healthy emmetropes (18 women). Because endothelial parameters differ between various populations, study of normal data from each population is essential [30]. Thus, the inclusion criteria consisted of healthy adult emmetropic subjects (mean spherical equivalent was -0.25 ± 0.75 diopters). Mean age was 20 ± 1.40 years, and their age range was 18–23 years. Comprehensive anterior segment examinations of all subjects were conducted using slit lamp. The exclusion criteria were prior corneal and/or intraocular surgery, existing corneal and/or significant anterior segment disease (e.g. cataract, keratectasia, retinopathy, etc.), intraocular pressure (IOP) ≥ 21 mmHg, and dry eye as determined by tear break-up time. Subjects with systemic disease and those taking any kind of medication were also excluded. IOP and corneal curvatures were determined by autorefractometry (Auto Kerato-Refracto-Tonometer TRK-1P; Topcon Corporation, Tokyo, Japan). The purpose of the study was explained to all subjects and informed consent was obtained from each one before beginning the examination. The study was conducted in conformance with the ethical considerations laid out in the 2008 Declaration of Helsinki, and the study protocol was approved by the research ethics review board of the College of Applied Medicine Science at King Saud University.

For each subject, one eye was selected randomly using a table of random numbers generated using Microsoft Excel (Microsoft Corporation, Redmond, WA, USA). Observer 1 conducted all measurements of the CCT, ECD, and CV using USP, SP-2000P, and z-ring CS4, while observer 2 conducted measurements of the CCT, ECD, and CV using z-ring CS4. The area of interest was standardized by 50 contiguous cells for SP-2000P and z-ring CS4 for ECD and CV measurements. Because it is known that CCT has been shown to increase overnight and return to baseline within 3 hours of waking, all the measurements of the CCT, ECD, and CV were collected during the afternoon between 12:00 noon and 2:00 pm.

Three consecutive measurements were performed with each technique for each subject by the two investigators. To diminish possible confounding factors caused by prior CCT measurement with another technique, a 30 minute interval was part of the protocol between taking measurements with the last two instruments. All the measurements were conducted in the same clinic at one location.

### Non-contact specular microscopy method

The method for using the non-contact specular microscope Topcon SP-2000P (Topcon Corp., Tokyo, Japan) was as follows. The subject’s head was positioned against the head band and chin rest, and they were then instructed to look straight ahead into the fixation targets. Pictures were captured of the central cornea area using the automatic-mode low flash intensity. Each image was taken after proper positioning of the alignment dot, circle, and bar on the screen. Endothelial cell morphology analyses were performed using automated measurements with the retracing method using the IMAGEnet® software program (Topcon), the manufacturer’s built-in image analysis software [31]. The estimated ECD was the mean of the three consecutive measurements, expressed as the number of cells per mm2. The images were printed with the analyzed data.

### USP method

For the USP (PacScan300p; Sonomed Inc., New York, USA) measurements, the probe was first disinfected with an alcohol swab. The seated subject was instructed to fixate on a distant target. One drop of a topical anesthetic (benoxinate hydrochloride 0.4%) was instilled in the conjunctival fornix of the eye to be tested. The ultrasound probe was then aligned perpendicular to the center of the cornea and placed gently in contact with the cornea. Three consecutive readings were taken for each subject.

### Confocal microscopy method

For the confocal microscopy (Confoscan 4; Nidek Technologies, Inc., Padova, Italy) measures, the subject’s head was placed on the head and a chin rest, and they were then instructed to look straight ahead at the fixation targets. One drop of a topical anesthetic (benoxinate hydrochloride 0.4%) was instilled in the eye. The tip of the objective lens and z-ring were covered with a viscous contact solution (GenTeal Gel®; Novartis Ophthalmics, East Hanover, NJ, USA), and the objective moved forward until the viscous contact solution contacted the cornea. The examiner adjusted the position of the objective lens until the field of view of the microscope was centered on the bright reflex from the endothelium and then pressed a button to begin a scan. After advancing past the anterior surface of the cornea, the microscope moved the focal plane to the original position and the scan was repeated. Cell analyses were conducted using the automated option of the NAVIS software (Confoscan 4 package version 3.6.6; Nidek Technologies, Inc., Padova, Italy).

Objective automated instruments use image-processing technology to recognize and analyze the cell characteristics data in confocal images, which improves the instruments’ repeatability and requires less analysis time [29,30]. Nevertheless, the primary disadvantage of confocal microscopy is that it is more challenging to perform, because of the involuntary movement of cells due to pulse, respiration, or ocular microsaccades, which can cause blurring of the detected images [32,33]. Each image-processing program developed has unique optical properties for each specular microscope instrument based on the cell selection criteria of the program. Thus, the images developed by a specific specular microscope instrument cannot be processed or analyzed by other specular microscope instrument image-processing programs [25].

### Statistical methods

The demographic data for all subjects were analyzed using Microsoft Excel 2007. InStat statistical software version 3.06 (GraphPad Software Inc., La Jolla, CA, USA) and Medcalc software version 11.4.4.0 were used for further statistical analyses. Repeated-measures analysis of variance (ANOVA) was conducted to compare ECD, CV, and CCT measurements. Bland–Altman analyses were performed to determine the repeatability of measurements for each instrument and to assess the limits of agreement between the three instruments. The paired *t*-test and unpaired *t*-test were used to test the differences in statistical significance. The level of statistical significance for this study was set at 0.05.

## Results

The study included 30 normal subjects (17 right eyes and 13 left eyes). The mean ± standard deviation (SD) IOP was 14.00 ± 1.50 mmHg. Two subjects dropped out of the study, as they were apprehensive about being examined with the z-ring CS4, thus they were excluded.

### Intra-observer repeatability, inter-observer reproducibility, and agreement between instruments in measuring ECD

The intra-observer repeatability analyses for the measurement of ECD using SP-2000P and z-ring CS4 were not statistically significant (ANOVA ≥ 0.05 and p = 0.13, observers 1 and 2, respectively), indicating a high repeatability of ECD measured with the SP-2000P and z-ring CS4.

The inter-observer reproducibility of ECD measured using the z-ring CS4 was not statistically significant, which indicates there to be high instrument reproducibility. Bland–Altman analysis of the mean difference for SP-2000P vs. z-ring CS4 (observer 1) showed good levels of agreement. The un-paired *t*-test revealed no significant difference (p = 0.46), which indicates that the two instruments can be used interchangeably. The mean ± SD, mean differences, limits of agreement and p-values for ECD measurements using the SP-2000P and z-ring CS4 are summarized in Table 1.

**Table 1:** **The means and standard deviations for ECD measurements obtained using the SP-2000P and z-ring CS4, intra-observer repeatability, inter-observer reproducibility (z-ring CS4 only), and comparison between SP-2000P vs. z-ring CS4 (mean differences, limits of agreement, paired *t*-test, un-paired test, and p-values)**

**Analysis of ECD values**	**Observer 1**		**Observer 2**
	**SP-2000P**	**z-ring CS4**	**z-ring CS4**
Mean ± SD*	3115.50 ± 279.70	3167.50 ± 264.75	3192.63 ± 249.42
Intra-observer repeatability (p-value)	0.22	0.99	0.13
	Mean difference ± SD*	Limits of agreement	p-value
Inter-observer reproducibility with z-ring CS4**	25.10 ± 128.92	+304, -354.20	0.30
Comparison between SP-2000P vs. z-ring CS4†	-52.10 ± 97.90	+139.80, -243.90	0.46

*Standard deviation.

**Paired test.

†Unpaired test.

### Intra-observer repeatability, inter-observer reproducibility, and agreement between instruments in measuring CV

The intra-observer repeatability analyses for the measurement of CV using SP-2000P and z-ring CS4 (observer 1) were not statistically significant (ANOVA; > 0.05 [both]). Also, the repeatability analysis for CV measured by observer 2 using the z-ring CS4 was not statistically significant (p = 0.98); thus, indicating high repeatability of the two instruments.

The inter-observer reproducibility for measurement of CV using the z-ring CS4 was not statistically significant, demonstrating high instrument reproducibility. Bland–Altman analysis of the mean differences demonstrated a good level of agreement for observer 1 using both the SP-2000P and z-ring CS4. The un-paired *t*-test showed there to be no significant inter-instrument difference (p = 0.79): indicating that the SP-2000P and z-ring CS4 can be used interchangeably. The mean ± SD, mean differences, limits of agreement, and p-values of CV measurements using SP-2000P and z-ring CS4 are summarized in Table 2.

**Table 2:** **The means and standard deviations for CV measurements obtained using the SP-2000P and z-ring CS4, intra-observer repeatability, inter-observer reproducibility (z-ring CS4 only), and comparison between SP-2000P vs. z-ring CS4 (mean differences, limits of agreement, paired *t*-test, un-paired test, and p-values)**

**Analysis of CV values**	**Observer 1**		**Observer 2**
	**SP-2000P**	**z-ring CS4**	**z-ring CS4**
Mean ± SD*	27.12 ± 2.51	27.10 ± 2.41	27.17 ± 2.25
Intra-observer repeatability (p-value)	0.75	0.34	0.98
	Mean difference ± SD*	Limits of agreement	p-value
Inter-observer reproducibility with z-ring CS4**	-0.20 ± 1.20	+3.80, -4.20	0.30
Comparison between SP-2000P vs. z-ring CS4†	0.20 ± 1.45	+3.00, -2.70	0.79

*Standard deviation.

**Paired test.

†Un-paired test.

### Intra-observer repeatability, inter-observer reproducibility, and agreement between instruments in measuring CCT

The intra-observer repeatability results for CCT measured using USP, SP-2000P, and z-ring CS4 (observer 1) were not statistically significant (ANOVA; p > 0.05). This was similarly the case for observer 2 for measuring CCT with the z-ring CS4 (ANOVA; p = 0.54). These data indicate there to be high repeatability of all three instruments in the measurement of CCT.

The inter-observer reproducibility data for CCT measurements with the z-ring CS4 was not statistically significant, demonstrating good instrument reproducibility. Bland–Altman analyses of the mean differences demonstrate poor levels of agreement for observer 1 using USP vs. SP-2000P, and SP-2000P vs. z-ring CS4, but demonstrate good levels of agreement for observer 1 using USP vs. z-ring CS4. The post hoc power result indicates that the CCT measurements in the current study has only 40% power (i.e. 60% type II error) to detect a difference in the present observed means (USP = 555.11, SP-2000P =535.82, z-ring CS4 = 552.57). This demonstrate that the sample size was too small to test for differences of the CCT measurements between the three instruments. The mean ± SD, mean differences, limits of agreement, and p-values of CCT measurements using the three instruments are summarized in Table 3.

**Table 3:** **The means and standard deviations for CCT measurements obtained using the SP-2000P and z-ring CS4, intra-observer repeatability, inter-observer reproducibility (z-ring CS4 only), and comparison between SP-2000P vs. z-ring CS4 (mean differences, limits of agreement, paired *t*-test, un-paired test, and p-values)**

**Analysis of CCT values**	**Observer 1**			**Observer 2**
	**USP**	**SP-2000P**	**z-ring CS4**	**z-ring CS4**
Mean ± SD*	555.11 ± 35.83	535.82 ± 41.10	552.57 ± 36.83	554.97 ± 36.34
Intra-observer repeatability (p-value)	0.91	0.20	0.45	0.54
	Mean difference ± SD*		Limits of agreement	p-value
Inter-observer reproducibility with z-ring CS4**	-2.40 ± 9.10		+39.90, -38.70	0.16
USP vs. SP-2000P	19.30 ± 19.60		+57.70, -19.20	
USP vs. z-ring CS4	2.50 ± 5.70		+16.30, -11.20	
SP-2000P vs. z-ring CS4	-16.70 ± 18.60		+23.70, -57.20	

Standard deviation*.

Paired test**.

## Discussion

Reproducible and repeatable objective means to evaluate corneal endothelial morphology and CCT are vital, in both clinical and research settings. Assessment of the endothelium is crucial to determine the suitability of donor corneas, to monitor the outcome of anterior segment procedures, such as cataract surgery, and when evaluating the safety of new intraocular or corneal surgical procedures and intraocular lenses [32,33]. Thus, assessment and validation of newly developed measurement techniques is essential to determine which techniques can be used interchangeably.

The results of this study indicate that the intra-observer repeatability of ECD, CV, and CCT measurements using z-ring CS4 were not significantly different (p ≥ 0.05). Also, there were no statistically significant differences in intra-observer repeatability for observer 1 of ECD, CV, and CCT measurements with SP-2000P and USP (CCT only). These results indicate a high reliability of repeat testing for ECD, CV, and CCT measurements using the three instruments.

The paired *t*-test p-values show that inter-observer reproducibility of the ECD, CV, and CCT measurements using z-ring CS4 were not statistically significantly different (p = 0.30, 0.30 and 0.16, respectively). Thus, the measurements of ECD, CV, and CCT taken with z-ring CS4 by observer 1 and observer 2 were in agreement. This means that the difference is acceptable and the inter-observer reproducibility on the same occasion were comparable.

The Bland–Altman analysis better shows the clinical relevance of repeatability between the three instruments. The measurements of ECD and CV by observer 1, using the SP-2000P and z-ring CS4, were in agreement; and the unpaired *t*-test p-values between the instruments were not significantly different (p = 0.74 and 0.25, respectively; Tables 1 and 2). This indicates that the difference between the instruments is comparable on the same occasion.

The post hoc power result indicates that the CCT measurements in the current study has only 40% power to detect a difference in the observed means; demonstrating that the sample size was too small to test for differences of the CCT measurements between the three instruments. However, Bland–Altman analyses of the mean differences indicate poor levels of agreement for observer 1 using USP vs. SP-2000P, and SP-2000P vs. z-ring CS4, and demonstrate good levels of agreement for observer 1 using USP vs. z-ring CS4.

It is vital to assess new instruments for reliability and to determine the agreement of results obtained using existing tools. Our results with SP-2000P and z-ring CS4 demonstrated not only reliable images for the assessment of ECD and CV, but also good repeatability and reproducible measurements of ECD, CV, and CCT—results that are supported by earlier studies [26,29,31,34-37]. Furthermore, in this study, results of the CCT measurements (using USP) showed good repeatability, which is supported by previous studies [26,29].

O’Donnell et al. (2004) reported the mean differences and p-values of the ECD and CV using the earlier non-contact specular microscope Topcon SP-1000P (SP-1000P) vs. the Confoscan 3 confocal microscope (CS3), which were 413 ± 163 cells/mm2 and 0.00001, and 1.45 ± 2.60% and 0.0003, respectively [28] [Table 4]. Their results indicate that there are statistically significant differences in measurements of ECD and CV between the two instruments and, thus, these instruments cannot be used interchangeably. This differs from our finding where the mean difference and unpaired *t*-test p-values of the ECD and CV with SP-2000P vs. z-ring CS4 were 52.10 ± 97.90 cells/mm2 (p = 0.46 [0.20 ± 1.45%] and p = 0.79, respectively). This could be because of the fact that the Topcon SP-2000P and z-ring CS4 are both upgraded versions of the SP-1000P and CS3. Other studies using one endothelial image that was read on two occasions independently by two examiners reported that the SP-1000P results show poor test-retest reliability [34,38]. Also, poor agreement between SP-1000P and CS3 to determine ECD is thought to be due to poor image quality [28]. In addition, one of the advantages of the z-ring CS4 that we used in our study is that the z-ring adapter is fixed in regard to the examined cornea; thus, it avoids the error produced by eye movements along the z-axis [29].

**Table 4:** Overview of recent studies comparing the repeatability and reproducibility of different instruments for measuring CCT, ECD and CV

**Author (year)**	**Instruments and parameters compared**		**Sample size**	**Age range**	**Clinical condition**	**Agreement between results?**
	**CCT**	**ECD/CV**				
**Cheung et al. (2000)**[29]		SP-2000P vs. IMAGEnet®	1.) 8 eyes to evaluate SP-2000P performance	20–29	Some subjects were contact lens wearers	No*
			2.) 7 eyes to evaluate reproducibility			
			3.) 12 eyes to evaluate repeatability			
**O’Donnell et al. (2004)**[28]		SP1000P vs. CS3	50 eyes	21–42	Neonates and contact lens wearers	No*
**Uçakhan et al. (2007)**[26]	USP vs. SP-2000P		45 eyes**/62 eyes†	13–52	Mild myopia and keratoconics	No*
**Brugin et al. (2007)**[32]	USP vs. z-ring CS4		44 eyes	22–49	22 eyes were post refractive surgery	No*

*In all studies the variation between instruments was statistically significant.

**Mild myopia (minus 1–6 dioptres).

†Patients with keratoconus.

Other studies have reported that the accuracy of the z-ring CS4 (in terms of repeatability and reproducibility for CCT measurements) is high [29,39]. Brugin et al. (2007) reported that the mean ± SD for USP and z-ring CS4 of the CCT measurements were 512.6 ± 65.8 μm and 487.80 ± 60.1 μm, respectively. The mean difference and p-value were 24.80 μm and 0.0001, respectively: indicating a statistically significant difference between the instruments [29] [Table 4]. Our results show that the z-ring CS4 gives high repeatability and reproducibility of CCT measurements, which is comparable with their study. However, our results of the post hoc power indicates that the CCT measurements of the current study has only 40% power to detect due to small sample size. Thus testing for differences of the CCT measurements between the three instruments will not provide sufficient statistical power. Although the use of younger patients between 18–23 years might be considered to be a study limitation, the aim of this study was to assess normal eyes and we considered that a younger group of patients would best represent this ideal sample, giving a homogenous patient population to test the validity of the instruments. Furthermore, in Saudi Arabia this sample is representative of younger patients who undergo refractive surgery prior to enrolling in university or committing to military service, so it is relevant to test the reproducibility and repeatability of z-ring CS4 in this group.

Another paper reported that the mean ± SD for USP and SP-2000P of the CCT measurements were 554.90 ± 7.40 μm and 535.50 ± 6.5 μm, respectively. The mean difference was 19.40 ± 14.7 μm and p < 0.01 [26] [Table 4]. Our findings are similar to their results the mean of the CCT measurements using USP and SP-2000P were 555.11 ± 35.83 μm and 535.41 ± 41.10 μm, respectively. The mean difference was 19.30 ± 19.60 μm, suggesting that SP-2000P were thinner than USP. In case of z-ring CS4 our results show that SP-2000P were thinner by 16.70 ± 18.60 μm. However, due to the smaller number of subjects leading to low statistical power conclusion can not be drawn.

## Conclusions

The SP-2000P and the z-ring CS4 can be used interchangeably to measure ECD and CV. For CCT, the sample size was too small to test for differences of the CCT measurements between the three instruments.

## Competing interests

The authors declare that they have no competing interests.

## Authors’ contributions

HA made a substantial contribution to conception and design, analysis and interpretation of data, drafting the manuscript and revising it critically for important intellectual content. WA was involved in study conception and design and analysis and interpretation of data. HR and AA participated in data acquisition. All authors read and approved the final manuscript.

## Pre-publication history

The pre-publication history for this paper can be accessed here:

http://www.biomedcentral.com/1471-2415/13/73/prepub
